# Effect of additional dimensions and views in the echocardiographic determination of 3‐dimensional left ventricular volume in myxomatous mitral valve disease in dogs

**DOI:** 10.1111/jvim.17300

**Published:** 2025-01-11

**Authors:** Weihow Hsue, Cortney E. Pelzek, Samantha Siess, Benjamin A. Terhaar, Shana B. Mintz, Romain Pariaut

**Affiliations:** ^1^ Department of Clinical Sciences College of Veterinary Medicine, Cornell University Ithaca New York USA

**Keywords:** 4D auto LVQ, area‐length, biplane, cube, real‐time triplane, Simpson's method of discs

## Abstract

**Background:**

Left ventricular (LV) volumes can be calculated from various linear, monoplane, and multiplane echocardiographic methods, and the same method can be applied to different imaging views. However, these methods and their variations have not been comprehensively evaluated against real‐time 3‐dimensional echocardiography (RT3D).

**Hypothesis/Objectives:**

To identify the LV volumetric approaches that produce the least bias and the best agreement with RT3D, and to assess interoperator reproducibility between an experienced and an inexperienced operator.

**Animals:**

Fifty‐nine client‐owned dogs with myxomatous mitral valve disease (38 Stage B1, 13 Stage B2, 8 Stages C/D) received echocardiograms, with a subset of 28 dogs (14 Stage B1, 10 Stage B2, 4 Stages C/D) imaged by 2 operators.

**Methods:**

Prospective method comparison study. Body weight‐indexed end‐diastolic and end‐systolic LV volumes using linear methods in long‐ and short‐axis views (Teichholz, cube, modified cube), monoplane methods in right parasternal and left apical views (area‐length and Simpson's method of discs), biplane Simpson's method of discs, and real‐time triplane (RT3P) were compared against RT3D.

**Results:**

The RT3P method exhibited no bias and demonstrated the highest agreement with RT3D. The linear methods showed significant bias and lower agreements for end‐diastolic volumes, end‐systolic volumes, or both. Volumes derived from different imaging views using the same method showed poor agreement. Both RT3P and RT3D methods demonstrated poor interoperator reproducibility.

**Conclusions and Clinical Importance:**

Incorporating additional dimensions improves bias and agreement in LV volume quantification, but comprehensive clinical experience with RT3P and RT3D is needed to improve consistency across all operators.

**Abbreviations**
1D: 1‐dimensional
2D: 2‐dimensional
3D: 3‐dimensional
ALM_A4C_
: left apical 4‐chamber area‐length method
ALM_RPL_
: right parasternal long‐axis 4‐chamber area‐length method
D3_Lx_
: long‐axis cube method
D3_Sx_
: short‐axis cube method
iEDV: body weight‐indexed end‐diastolic volume
iESV: body weight‐indexed end‐systolic volume
LV: left ventricle
mD3_Lx_
: long‐axis modified cube method
mD3_Sx_
: short‐axis modified cube method
MMVD: myxomatous mitral valve disease
MOD_2P_
: left apical biplane Simpson's method of discs
MOD_A4C_
: left apical 4‐chamber Simpson's method of discs
MOD_RPL_
: right parasternal long‐axis 4‐chamber Simpson's method of discs
MRI: magnetic resonance imaging
RT3D: real‐time 3‐dimensional echocardiography
RT3P: real‐time triplane
Tei_Lx_
: long‐axis Teichholz method
Tei_Sx_
: short‐axis Teichholz method

## 1 INTRODUCTION

Myxomatous mitral valve disease (MMVD) is the most common heart disease in dogs, often leading to progressive cardiac enlargement and eventual congestive heart failure. While left atrial size is emphasized for assessing prognosis and guiding treatment, the left atrium‐to‐aortic root ratio, the most utilized measurement, is susceptible to inconsistent reproducibility.^1^, ^2^ Evaluating left ventricular (LV) size thus serves as a complementary approach to ascertain cardiac enlargement, typically via normalized internal diameters.^3^ However, as interventional and surgical therapies become accessible, derivation of LV volumes to calculate mitral regurgitant fraction will be vital.^4^, ^5^ Currently, no universally accepted gold standard exists for quantifying LV volume in dogs, although 3‐dimensional (3D) modalities are potential candidates as they do not require geometric assumptions. Cardiac magnetic resonance imaging (MRI) is considered the most accurate in humans but requires anesthesia in dogs, which could negatively impact cardiac function. Real‐time 3D echocardiography (RT3D), which has been shown to correlate well with MRI, can be comfortably utilized in awake dogs with severe cardiac disease.^6^ However, like MRI, it is hindered by the need for specialized equipment (ie, dedicated transducers and software), accessibility, expertise, and costs, leaving daily practice still dependent on 1‐dimensional (1D) and 2‐dimensional (2D) echocardiography.

The 1D methods convert the linear LV internal diameter from the right parasternal short‐axis M‐mode view to LV volume using the Teichholz,^7^ cube,^8^ or modified cube^9^ formula, each of which assumes different geometric shapes. The LV internal diameter can also be obtained from the long‐axis view, which may be less susceptible to angling and thus more accurate.^10^ However, because the geometry of the LV changes as it enlarges, these formulas may not be applicable with chamber dilatation. In contrast, 2D monoplane methods, such as the area‐length method and the Simpson's method of discs, outline the chamber on a single plane and require fewer geometric assumptions. These can also be acquired from either the right parasternal long‐axis view or the left apical view, which are considered non‐interchangeable.^9^ Correspondingly, biplane and real‐time triplane (RT3P) methods, the latter of which is a simplified version of RT3D that simultaneously captures 3 2D long‐axis planes of the heart within a single cardiac cycle,^11^ may further improve accuracy by incorporating additional dimensions and minimizing geometric assumptions. However, these more complex methods may present a steeper learning curve, posing challenges in achieving consistent and reproducible results across operators with varying levels of experience.

Understanding which methods and variations best approximate RT3D estimates of LV volume, along with evaluating their interoperator reproducibility (ie, the variation when different operators independently obtain and perform measurements using the same method on the same subject at the same time) between experienced and inexperienced operators, is of clinical interest. Therefore, the objectives of this study are: (a) to assess bias and agreement of 1D, 2D, and RT3P echocardiographic estimates of LV volume against RT3D in dogs with MMVD; (b) to determine whether different imaging views of these methods affect their bias and agreement; and (c) to compare interoperator reproducibility between an experienced cardiologist and resident‐in‐training.

## 2 MATERIALS AND METHODS

All procedures were approved by the Institutional Animal Care and Use Committee at Cornell University (protocol #: 2022‐0218). All dog owners provided written, informed consent before enrollment.

### 2.1 Animals and experimental design

This was a single‐center, prospective method comparison study conducted from December 2022 to October 2023. Client‐owned dogs presenting to the Cornell University Hospital for Animals were recruited. To be included, dogs had to be ≥6 years of age, have a characteristic left apical systolic murmur, and have echocardiographic confirmation of MMVD (valve thickening, irregularity, and/or prolapse with presence of mitral regurgitation on color Doppler). Dogs were permitted to receive medications affecting the cardiovascular system if they had been started more than 7 days before examination. Exclusion criteria included the presence of any other cardiovascular disease, significant arrhythmias, or the need for sedation during imaging.

All echocardiographic examinations and measurements were performed by a board‐certified cardiologist (W.H), who routinely uses 1D and 2D methods in clinical practice and has experience with RT3P and RT3D methods in multiple clinical research projects. In a subset of dogs, echocardiographic exams were also separately performed by a cardiology resident‐in‐training (C.E.P.). The cardiologist did not work at the same institution until the resident's second year of training when the study commenced, so the resident's first year of echocardiographic experience was independent of the cardiologist. The resident regularly uses 1D and 2D methods in clinical practice but received training in RT3P and RT3D under the cardiologist's guidance for the purposes of this study. The subset of dogs was chosen based on clinical convenience, when both operators were simultaneously available to perform back‐to‐back echocardiograms. When 2 exams were performed, the sequence of operator was determined by simple randomization (ie, coin toss). Both operators performed their own measurements on their respective images and were masked from the measurements of the other operator.

### 2.2 Echocardiographic examination and measurements

Routine M‐mode, 2D, and Doppler imaging were performed with a Vivid E95 ultrasound unit (GE Healthcare, Chicago, Illinois) equipped with several phased‐array transducers (5‐12 MHz) tailored to the size of the dog. The RT3P and RT3D images were acquired using the 6Vc‐4D and 4Vc‐4D transducer, respectively. Simultaneous electrocardiographic monitoring was employed. Recommended tomographic imaging planes were obtained, including the right parasternal long‐axis 4‐chamber view, the right parasternal short‐axis view at the level of the papillary muscle, the left apical 4‐chamber view, and the left apical 2‐chamber view.^12^ Additionally, the left apical 3‐chamber view, which simultaneously visualizes the mitral valve and LV outflow tract, was acquired as part of RT3P and RT3D.^11^, ^13^ Both end‐diastolic and end‐systolic volumes were obtained; end‐diastole was defined as the frame immediately following mitral valve closure, and end‐systole as the frame with the smallest LV cavity immediately before mitral valve opening. Because the mitral valve could not be visualized on M‐mode, end‐diastole on M‐mode was defined as the onset of the QRS complex on ECG and end‐systole as the frame with the smallest cavity size. All measurements were averaged over 3 to 5, usually consecutive, cycles, allowing the operator the choice of including additional cycles if motion or respiratory motion impacted image consistency. Because of the blinded nature of the study, efforts to standardize heart rate were not pursued. The 1D, 2D with RT3P, and 3D measurements were obtained from separate offline sessions using dedicated software (EchoPac, GE Healthcare, Chicago, Illinois), with operators blinded to any previous results. All volumetric measurements were divided by body weight to obtain indexed end‐diastolic volumes (iEDV) and indexed end‐systolic volumes (iESV).

All 1D methods utilized the LV internal diameter derived from the right parasternal long‐axis 4‐chamber view or the right parasternal short‐axis view at the level of the papillary muscles. On the long‐axis view, the LV internal diameter was measured at the chordal level roughly perpendicular to the long‐axis of the interventricular septum and LV free wall as previously described using an inner edge‐to‐inner edge technique.^14^ The distance was then used to derive LV volume via the Teichholz (Tei_Lx_),^7^ cube (D3_Lx_),^8^ and modified cube (mD3_Lx_ = 0.67 × D3_Lx_)^9^ methods. On the short‐axis view, the same LV internal diameter was obtained on M‐mode using the inner edge‐to‐inner edge technique, and LV volume was similarly calculated via the Teichholz (Tei_Sx_), cube (D3_Sx_), and modified cube (mD3_Sx_ = 0.67 × D3_Sx_) methods.

The 2D methods consisted of monoplane and biplane modalities. All volumes were calculated based on manual tracing of the LV endocardial border, including the papillary muscles, with a straight line from hinge point to hinge point across the mitral valve defining the basilar border of the LV. A straight line from the midpoint of the mitral valve annulus to the apex defined the length of the LV. Monoplane volumes were derived using the area‐length method and the Simpson's method of discs as previously described on the right parasternal long‐axis 4‐chamber views (ALM_RPL_ and MOD_RPL_) and the left apical 4‐chamber view (ALM_A4C_ and MOD_A4C_).^15^, ^16^, ^17^ Biplane volumes were derived using the Simpson's method of discs on the left apical 4‐chamber and 2‐chamber views (MOD_2P_).^16^

**FIGURE 1:**
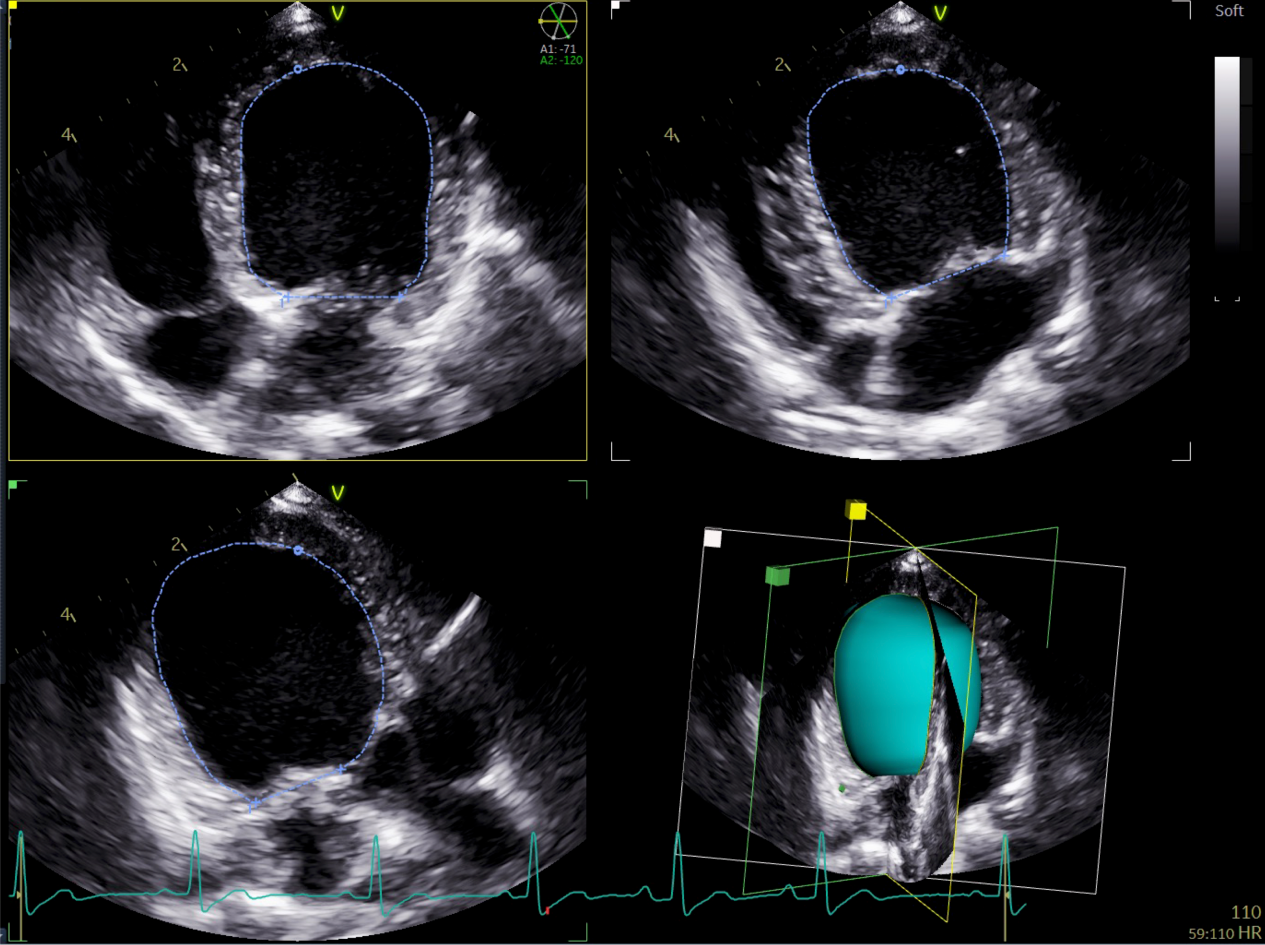
Real‐time triplane view and measurement of left ventricular end‐diastolic volume. The 4‐chamber view is in the top left corner, the 2‐chamber view is in the top right corner, the 3‐chamber view is in the bottom left corner, and the resulting 3‐dimensional estimated shell is in the bottom right corner. The software allows manual tracing of the cavity in each imaging plane to calculate the volume.

The RT3P images were acquired and measured from 3 simultaneous planes, with the left apical 4‐chamber view serving as the primary reference. The 2‐chamber and 3‐chamber views were displayed at inter‐plane angles set to 60°, with manual adjustments as needed to minimize foreshortening (Figure 1). The RT3P volumes were computed by the software via surface triangulation and summation of all triangles by use of the divergence theorem.^11^, ^15^

The RT3D images consisted of real‐time multiplane views of the LV with multi‐beat acquisition, with a target frame rate of >30 frames per second. The same 3 left apical views for RT3P, along with short‐axis visualization of the LV cavity at 3 different levels, were optimized during acquisition (Figure S1). The RT3D volumes were calculated using the semi‐automated 4D Auto LVQ volume quantification module.^13^ In the initial quad‐screen, 3 apical views with 60° interplane spacing (similar to RT3P) and a short‐axis view are seen. Manual adjustments can be made at this step to optimize visualization of standard imaging planes with minimal foreshortening (4‐chamber, 2‐chamber, 3‐chamber views). End‐diastolic images are shown on the following screen, with the option to adjust the exact end‐diastolic timing if necessary. Automated surface detection is initialized in this screen by manually selecting the LV apex and mitral annulus. The borders of the LV can then be manually adjusted in any long‐axis or short‐axis frame, including all intermediate frames between the standard planes (Video S1). The procedure is then repeated for end‐systole on the following screen. After completed surface tracing, RT3D volumes were derived from the triangulated surfaces by summation of all triangular patches using the divergence theorem.

### 2.3 Statistical analysis

All statistical analyses, including quantification of reproducibility parameters, were performed using MedCalc (MedCalc Software Ltd, Ostend, Belgium). Normality was assessed with the Shapiro‐Wilk test. Quantitative data were expressed as median (2.5th‐97.5th percentile) unless otherwise specified. *Bias* and *agreement* were analyzed when comparing different measurement methods to RT3D and between different views of the same method. *Bias* was defined as a non‐zero median of the differences between 2 methods, while agreement, defined as the degree of consensus between 2 sets of measurements, was evaluated using Lin's concordance correlation coefficient.^18^, ^19^ The concordance correlation coefficient accounts for both the Pearson correlation coefficient, a measure of the deviation from the best‐fit line, and a bias correction factor, a measure of the deviation of the best‐fit line from the 45° line through the origin. Values of <0.90 indicated poor agreement, 0.90‐0.95 indicated moderate agreement, 0.95‐0.99 indicated substantial agreement, and >0.99 indicated almost perfect agreement.^19^ In addition, Bland‐Altman plots were generated to visualize the line of equality, regression line of differences, and limits of agreement.^20^ Significance was set at 2‐sided *α* = 0.05.

Interoperator reproducibility was evaluated using coefficients of variation (which quantify the differences relative to the mean), intraclass correlation coefficients (which quantify the association between different operators), and 95% reproducibility coefficients (which quantify the limits between which 95% of repeated measures are expected to fall, expressed in the same units as the measurement of interest).^21^ Coefficients of variation were derived using the within‐subject SD and were calculated as: (within‐subject SD/overall mean) × 100. The intraclass correlation coefficients were calculated using a 2‐way model with absolute agreement and were reported by single measures; values <0.5 indicated poor reproducibility, 0.5‐0.75 indicated moderate reproducibility, 0.75‐0.90 indicated good reproducibility, and >0.90 indicated excellent reproducibility.^22^ The 95% reproducibility coefficients were calculated as 1.96 × SD of the differences between 2 sets of measurements.^2^, ^23^

## 3 RESULTS

Echocardiography was performed in 64 dogs. Four dogs were excluded because of poor image quality that precluded adequate 3D measurement. An additional dog, a whippet, was excluded as an outlier because its LV volumes greatly exceeded the upper 95 percentile range of the remaining dogs by a factor of up to 5, even after verifying the accuracy of the measurements. For instance, its iESV via mD3_Sx_ and RT3D were 3.66 and 4.08 mL/kg, respectively, while the corresponding medians (2.5th‐97.5th percentile) of the remaining dogs were 0.39 (0.15‐0.72) and 0.64 (0.26‐1.21) mL/kg, respectively. All median LV volumes, both raw and body weight‐indexed, of the 59 dogs included in the analysis are presented in Table 1. Nineteen breeds were represented, including Cavalier King Charles Spaniel (20), mixed breed (14), Chihuahua (4), Beagle (2), Dachshund (2), Maltese (2), Miniature Schnauzer (2), Shih Tzu (2), and 1 each of Boston Terrier, Dalmatian, German Shorthaired Pointer, Havanese, Jack Russell Terrier, Labrador Retriever, Pekingese, Pomeranian, Red Bone Hound, Small Münsterländer, and Yorkshire Terrier. Thirty‐seven (63%) were male and 22 (37%) were female. The median age was 11 (range, 6‐16) years, and the median weight was 8.4 (range, 3.6‐39.2) kg. According to the American College of Veterinary Internal Medicine consensus statement on MMVD, 38 dogs were classified as Stage B1, 13 as Stage B2, 7 as Stage C, and 1 as Stage D.^3^

**TABLE 1:** Median (2.5th‐97.5th percentile) of raw LV volumes and body weight‐indexed LV volumes from all echocardiographic methods.

Method		End‐diastolic volume (mL)	End‐systolic volume (mL)	iEDV (mL/kg)	iESV (mL/kg)
1D	Tei_Lx_	34.72 (13.51‐96.42)	8.26 (2.05‐36.44)	3.90 (1.98‐7.75)	0.97 (0.41‐1.77)
	Tei_Sx_	36.44 (13.19‐117.46)	8.91 (1.31‐39.02)	4.15 (1.43‐9.36)	1.01 (0.26‐1.99)
	D3_Lx_	26.73 (8.59‐96.20)	4.83 (1.00‐28.37)	3.18 (1.33‐6.34)	0.58 (0.22‐1.07)
	D3_Sx_	28.37 (8.35‐123.94)	5.27 (0.60‐30.86)	3.33 (0.90‐7.76)	0.62 (0.12‐1.39)
	mD3_Lx_	17.91 (5.75‐64.46)	3.23 (0.67‐19.01)	2.13 (0.89‐4.25)	0.39 (0.15‐0.72)
	mD3_Sx_	19.01 (5.59‐83.04)	3.53 (0.41‐20.68)	2.23 (0.60‐5.20)	0.42 (0.08‐0.93)
2D	ALM_RPL_	23.97 (9.19‐95.20)	5.67 (1.45‐33.06)	2.58 (1.60‐5.09)	0.64 (0.25‐1.30)
	ALM_A4C_	25.17 (10.52‐89.04)	5.43 (1.62‐33.09)	2.78 (1.55‐5.45)	0.72 (0.33‐1.30)
	MOD_RPL_	24.62 (9.23‐88.43)	5.36 (1.81‐34.77)	2.71 (1.58‐5.01)	0.65 (0.26‐1.19)
	MOD_A4C_	24.84 (9.71‐94.74)	5.77 (1.75‐31.76)	2.68 (1.48‐5.18)	0.75 (0.35‐1.31)
	MOD_2P_	26.08 (10.53‐82.82)	6.18 (1.68‐28.21)	2.94 (1.75‐5.31)	0.69 (0.37‐1.39)
RT3P		22.66 (10.14‐85.94)	5.98 (1.72‐30.86)	2.63 (1.36‐4.46)	0.70 (0.24‐1.14)
RT3D		22.80 (8.88‐84.03)	5.20 (1.60‐35.70)	2.58 (1.24‐4.51)	0.64 (0.26‐1.21)

The median of the differences and the concordance correlation coefficient for each 1D, 2D, and RT3P method compared to RT3D are detailed in Table S1.

**FIGURE 2:**
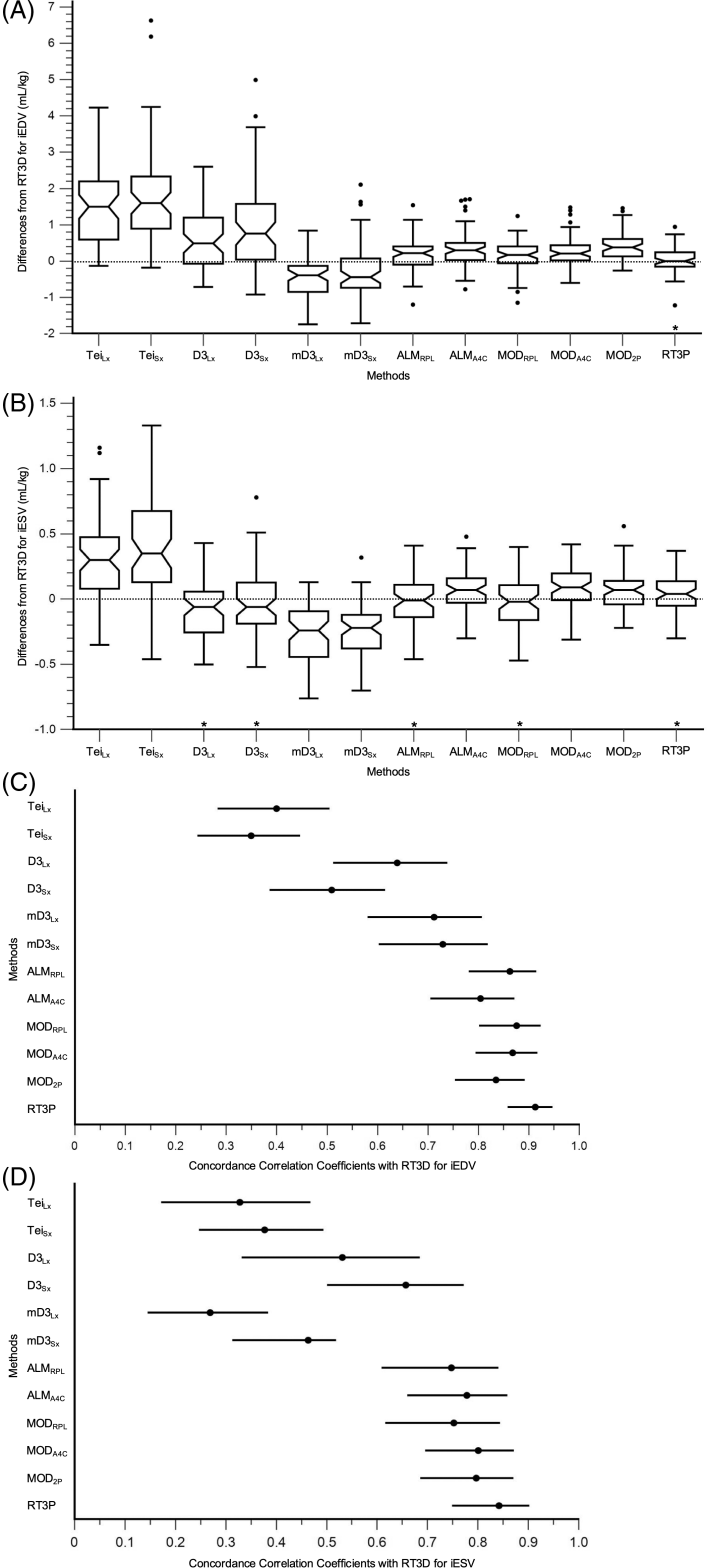
Bias and agreement of indexed LV volumes for each 1D, 2D, and RT3P echocardiographic method compared to RT3D. Box‐and‐whisker plots of the differences between each method and RT3D are shown for iEDV (A) and iESV (B). The notched areas outline the 95% confidence interval for the median of the differences. If this interval overlaps zero, then the corresponding method is considered to have no systematic bias with RT3D (marked by *). Additionally, forest plots of the concordance correlation coefficients and corresponding 95% confidence intervals between each method and RT3D are shown for iEDV (C) and iESV (D). Significant differences are determined when there is no overlap between intervals.

**FIGURE 3:**
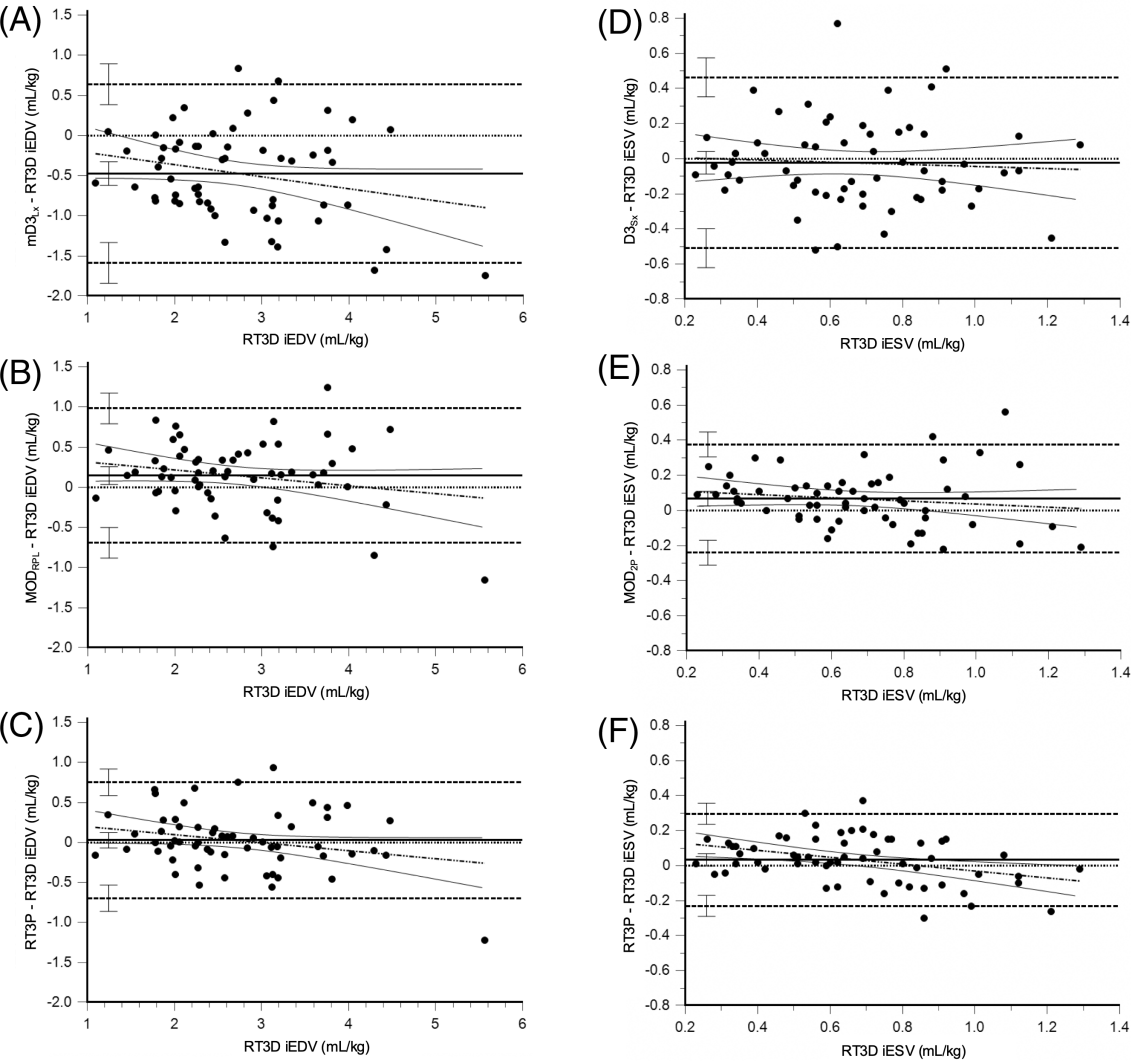
Bland‐Altman plots comparing selected 1D, 2D, and RT3P methods with RT3D. The 1D and 2D methods with the least systematic/proportional bias and smallest limits of agreement, along with RT3P, are shown for iEDV (A‐C) and iESV (D‐F). The line of equality (horizontal solid line), regression line of differences (alternating thick and thin dotted line), limits of agreement (horizontal thick dotted lines), and their 95% confidence intervals are displayed. Bland‐Altman plots for all methods are shown in Figures S2 and S3.

For iEDV, only RT3P had a median of the differences that included zero within its 95% confidence interval, indicating no systematic bias (Figure 2A). Except for mD3_Lx_ and mD3_Sx_, all remaining methods overestimated RT3D LV volume, with Tei_Lx_ and Tei_Sx_ having significantly greater bias. On the Bland‐Altman plots, the mD3_Lx_, mD3_Sx_, all 2D, and RT3P methods had regression slopes that were not significantly different than zero (mD3_Lx_: *P* = .07, mD3_Sx_: *P* = .53, ALM_RPL_: *P* = .19, ALM_A4C_: *P* = .60, MOD_RPL_: *P* = .12, MOD_A4C_: *P* = .83, MOD_2P_: *P* = .48, RT3P: *P* = .07; Figure 3A‐C; Figure S2). The remaining Teichholz and cube methods had larger positive bias at larger volumes (ie, positive proportional bias). According to the concordance correlation coefficient, RT3P was the only method to achieve at least moderate agreement with RT3D (*r*_*c*_ = 0.913 [0.858‐0.947]). The agreements of Tei_Lx_, Tei_Sx_, D3_Lx_, and D3_Sx_ were significantly lower than those of the 2D and RT3P methods, except for between D3_Lx_ and ALM_A4C_ (Figure 2C).

For iESV, the D3_Lx_, D3_Sx_, ALM_RPL_, MOD_RPL_, and RT3P methods had medians of the differences that included zero within their 95% confidence interval, indicating the potential for no systematic bias (Figure 2B). Except for mD3_Lx_, and mD3_Sx_, all remaining methods overestimated RT3D LV volume, with Tei_Lx_, Tei_Sx_, mD3_Lx_, and mD3_Sx_ having significantly greater bias. On the Bland‐Altman plots, only the Tei_Lx_ (*P* = .06), Tei_Sx_ (*P* = .11), D3_Sx_ (*P* = .64), ALM_A4C_ (*P* = .34), MOD_A4C_ (*P* = .64), and MOD_2P_ (*P* = .28) methods had regression slopes that were not significantly different than zero (Figure 3D‐F; Figure S3). The remaining D3_Lx_, modified cube, ALM_RPL_, MOD_RPL_, and RT3P methods had larger negative bias at larger volumes (ie, positive proportional bias). The concordance correlation coefficient indicated that all methods demonstrated poor agreement with RT3D, although RT3P showed the highest agreement (*r*_*c*_ = 0.842 [0.749‐0.902]). The agreements of Tei_Lx_, Tei_Sx_, mD3_Lx_, and mD3_Sx_ were significantly lower than those of the 2D and RT3P methods (Figure 2D).

Among the methods applicable to different imaging views, all 1D methods had 95% confidence intervals for the medians of the differences that narrowly included zero, indicating a potential lack of bias between long‐axis and short‐axis views (Table 2). However, all methods, both 1D and 2D, showed poor agreement between different views. Overall, the short‐axis and the left apical views trended toward larger LV volumes than the long‐axis views for the 1D methods and the right parasternal views for the 2D monoplane methods, respectively.

**TABLE 2:** Bias and agreement of indexed LV volumes between the same echocardiographic methods from different imaging views.

		iEDV					iESV				
		Differences		Concordance correlation			Differences		Concordance correlation		
Method		Median (95% CI)	2.5th‐97.5th percentile	*r*_*c*_ (95% CI)	*r*_*p*_	*C*_*b*_	Median (95% CI)	2.5th‐97.5th percentile	*r*_*c*_ (95% CI)	*r*_*p*_	*C*_*b*_
1D	Tei_Sx_ − Tei_Lx_	0.35 (0.00‐0.56)	−1.30‐1.98	0.887 (0.826‐0.927)	0.916	0.968	0.08 (0.00‐0.14)	−0.48‐0.64	0.769 (0.664‐0.845)	0.827	0.931
	D3_Sx_ − D3_Lx_	0.32 (0.00‐0.52)	−1.04‐1.80	0.861 (0.791‐0.908)	0.907	0.949	0.05 (0.00‐0.09)	−0.34‐0.50	0.751 (0.642‐0.831)	0.821	0.915
	mD3_Sx_ − mD3_Lx_	0.22 (0.00‐0.40)	−0.70‐1.21	0.860 (0.791‐0.908)	0.907	0.949	0.04 (0.00‐0.06)	−0.23‐0.33	0.750 (0.640‐0.830)	0.820	0.915
2D	ALM_A4C_ − ALM_RPL_	0.12 (0.06‐0.29)	−0.69‐1.44	0.871 (0.797‐0.919)	0.894	0.974	0.05 (0.02‐0.10)	−0.19‐0.49	0.782 (0.668‐0.860)	0.825	0.948
	MOD_A4C_ − MOD_RPL_	0.13 (0.01‐0.25)	−0.55‐0.89	0.876 (0.802‐0.924)	0.888	0.987	0.08 (0.04‐0.15)	−0.11‐0.54	0.733 (0.607‐0.823)	0.811	0.905

Abbreviations: *C*_*b*_, bias correction factor; CI, confidence intervals; *r*_*c*_, concordance correlation coefficient; *r*_*p*_, Pearson correlation coefficient.

**FIGURE 4:**
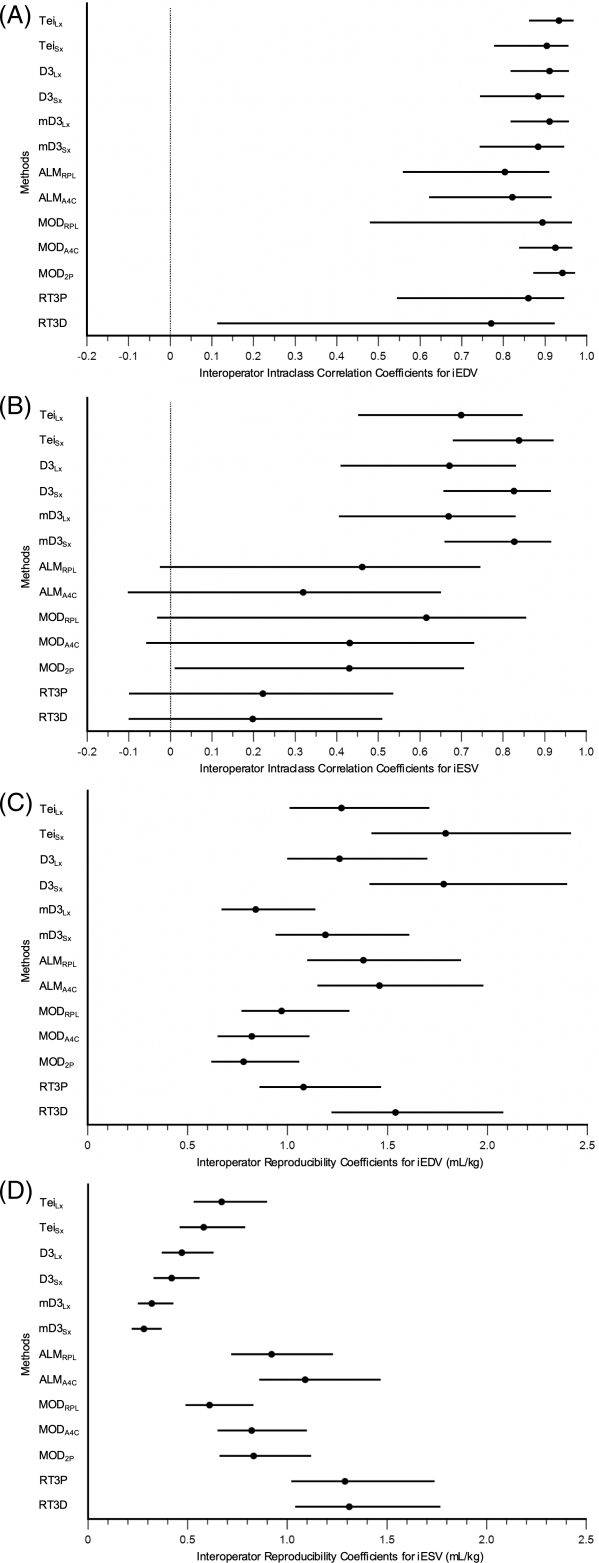
Interoperator intraclass correlation coefficients and reproducibility coefficients of all echocardiographic methods. Forest plots of the intraclass correlation coefficients and corresponding 95% confidence intervals for each echocardiographic method are shown for iEDV (A) and iESV (B). Additionally, forest plots of the reproducibility coefficients and corresponding 95% confidence intervals for each echocardiographic method are shown for iEDV (C) and iESV (D). Significant differences are determined when there is no overlap between intervals.

A subset of 28 dogs (14 Stage B1, 10 Stage B2, 3 Stage C, 1 Stage D) was imaged by 2 operators to evaluate interoperator reproducibility. Their coefficients of variation are presented in Table 3, and the intraclass correlation coefficients and reproducibility coefficients are detailed in Table S2. The iEDVs generally had coefficients of variation between 8% and 18% and good to excellent intraclass correlation coefficients across all methods, although none were significantly different from one another because of wide confidence intervals (Figure 4A). The lowest reproducibility coefficients were observed with MOD_A4C_ and MOD_2P_, which were significantly lower than those of Tei_Sx_, D3_Sx_, ALM_A4C_, and RT3D (Figure 4C). The iESVs generally had coefficients of variation between 19% and 49% and poor to moderate intraclass correlation coefficients with wide confidence intervals, except for Tei_Sx_, D3_Sx_, and mD3_Sx_ (Figure 4B). The lowest reproducibility coefficients were observed with mD3_Lx_ and mD3_Sx_, which were significantly lower than those of Tei_Lx_, Tei_Sx_, all 2D methods, RT3P, and RT3D (Figure 4D). Notably, RT3D had significantly larger reproducibility coefficients than mD3_Lx_, MOD_A4C_, and MOD_2P_ for iEDV, and larger reproducibility coefficients than all 1D methods and MOD_RPL_ for iESV. Similarly, the RT3P method had significantly larger reproducibility coefficients than all 1D methods and MOD_RPL_ for iESV.

**TABLE 3:** Coefficients of variation of all echocardiographic methods.

		Coefficient of variation	
Method		iEDV	iESV
1D	Tei_Lx_	10.1	23.2
	Tei_Sx_	12.9	19.6
	D3_Lx_	12.5	26.7
	D3_Sx_	15.4	22.5
	mD3_Lx_	12.5	26.8
	mD3_Sx_	15.4	22.4
2D	ALM_RPL_	15.7	40.6
	ALM_A4C_	15.2	39.8
	MOD_RPL_	11.2	29.5
	MOD_A4C_	9.1	30.9
	MOD_2P_	8.4	33.1
RT3P		12.8	48.7
RT3D		17.4	48.6

## 4 DISCUSSION

The RT3P was the method that best approximated RT3D, as it was the only technique without systematic bias and achieved the highest agreement for both iEDV and iESV. When real‐time multiplane imaging is unavailable, 2D methods are preferred over 1D methods because of notable limitations of the latter. Specifically, the Teichholz methods yielded significant bias and poor agreement across all volumes, the cube methods demonstrated poor agreement for iEDV, and the modified cube methods revealed significant bias and poor agreement for iESV. Imaging views from the same method did not significantly alter bias and agreement with RT3D. However, because volumes obtained from different views showed poor agreement with each other, it is still recommended to derive LV volume consistently from the same imaging view. Finally, RT3P and RT3P had lower interoperator reproducibility than many of the other methods, suggesting a potentially steeper learning curve for these methods.

The RT3P's superiority in estimating RT3D likely stems from its ability to employ multiple imaging planes within the same cardiac cycle, thus requiring fewer geometric assumptions. To the authors' knowledge, RT3P has only been previously evaluated in healthy Beagles in veterinary studies,^15^ where, similar to human studies,^11^ it demonstrated high correlation with MRI and low interobserver variability. However, our iESV measurements showed slight negative bias at larger volumes and poor agreement with RT3D (though still the best among all methods). This may reflect the increased difficulty in discerning endocardial borders from papillary muscles in small cavity sizes across multiple planes during end‐systole. Interestingly, unlike RT3P, MOD_2P_ did not improve agreement beyond that of the monoplane methods, possibly because the 2 planes were obtained from different cardiac cycles. In conclusion, RT3P shows promise as a simpler alternative for RT3D.

The results of this study align with the American Society of Echocardiography guidelines, which recommend 2D volumetric methods over 1D methods.^16^ The Teichholz methods exhibited significant bias and poor agreement regardless of imaging view, consistent with previous findings that the Tei_Sx_ method overestimates 3D volume in dogs with MMVD.^15^, ^24^, ^25^ Similarly, while the cube method was originally validated in canine cadaver hearts^26^ and has been used in various canine clinical studies,^8^, ^9^ its iEDV demonstrated significantly lower agreement than all 2D methods except for ALM_A4C_. Furthermore, although the modified cube method was previously found to better approximate volumes obtained by Simpson's method of discs than the cube method,^9^ it significantly underestimated iESV on RT3D. Proposed reasons for these poor performances include: (a) the formulas and correction factors were derived from conditions that do not simulate a beating canine heart; (b) asynchronous contraction^27^ and asymmetric chambers can invalidate these methods; (c) the geometry of the LV changes with progressive enlargement; and (d) cubing diameters may compound errors.

No imaging view consistently proved superior for any given method, as their indices of bias and agreement all overlapped. However, while the 1D methods may have no bias between the long‐axis and short‐axis views, their poor agreements undermine their interchangeability. The short‐axis 1D methods trended toward larger volumes than the long‐axis views, likely because of increased angling from the inability to confirm perpendicular alignment to the long‐axis of the heart. Similarly, the 2D left apical methods trended toward larger bias than their right parasternal counterparts, possibly because of differences in the likelihood for foreshortening. In conclusion, as with previous studies that evaluated both views,^9^, ^15^, ^28^, ^29^, ^30^, ^31^ the poor agreements imply that sonographers should adhere to the same method and imaging view for repeated measurements.

The RT3P and RT3D methods consistently had low interoperator reproducibility, especially for iESV, despite the resident receiving direct training from the cardiologist on these methods but not on the 1D or 2D methods. This contrasts with the traditionally high reproducibility associated with RT3D.^6^, ^24^, ^32^ Because endocardial tracing is semi‐automated, which should promote uniformity, the discrepancy is postulated to be from differences in the quality of image acquisition. The lower temporal and spatial resolution inherent in 3D echocardiography demands greater expertise for optimal image acquisition, which poses extra challenges for the small end‐systolic chamber sizes. Although multi‐beat acquisition improved resolution, the prominence of sinus arrhythmia and the inability to perform breath holds in awake dogs also introduced stitch artifacts. Additional factors, such as the emphasis on *interoperator* rather than *intraoperator* reproducibility and the inclusion of a broader range of disease severities, may have further contributed to the lower reproducibility compared to similar studies.^6^, ^24^ Future research should prioritize evaluating reproducibility among experienced operators.

Interoperator reproducibility among the 1D and 2D methods was more insightful, given that the operators were trained at different institutions yet routinely use these techniques in clinical practice. We report coefficients of variation because they are widely used in veterinary studies and serve as a dimensionless index that facilitates comparisons among different indices within the same sample population. Although sample populations differ and comparisons are flawed, our values were higher than those reported in other studies. This discrepancy may be because of differences in operator experience, the inclusion of healthy dogs versus those with cardiac disease,^6^, ^17^, ^29^, ^31^ or the fact that 2 operators independently obtained images and performed measurements.^17^, ^24^, ^28^, ^29^, ^31^, ^33^ We focused on *interoperator* reproducibility because it better reflects clinical practice, where different operators assess and follow up on cases. However, this naturally introduces greater variability than studies involving a single operator (*intraoperator*) or multiple observers measuring the same set of images (*interobserver*).^17^, ^24^ This study also reports intraclass correlation coefficients and reproducibility coefficients. The intraclass correlation coefficients had wide confidence intervals, so significant differences were not apparent; increasing the number of study subjects might mitigate this issue. The main advantage of reproducibility coefficients is that all methods in this study measured the same parameter using the same units, allowing for direct comparisons. The MOD_A4C_ and MOD_2P_ had the smallest reproducibility coefficients for iEDV, performing significantly better than the short‐axis 1D methods. The difficulty in obtaining a perfectly perpendicular short‐axis view likely accounts for their poor performance compared to the long‐axis methods. Interestingly, the mD3_Lx_ and mD3_Sx_ had the smallest reproducibility coefficients for iESV, significantly outperforming all 2D methods. A possible explanation is that, because of greater superimposition of the endocardial border and the papillary muscles at end‐systole, it is more challenging to separate them around the entire chamber as opposed to at just 2 points. In other words, simpler methods for iESV may trend toward lower variability (ie, better reproducibility) because there are fewer opportunities for variation to occur.

The major limitation of this study is that, although MRI is considered the gold standard for volumetric quantification in human medicine, RT3D was chosen as the reference standard. The use of MRI in veterinary echocardiographic studies is commonly limited by anesthetic risks and costs. In contrast, RT3D is performed similarly to routine echocardiography, facilitating noninvasive and cost‐effective evaluation of many dogs with varying degrees of cardiac disease severity. Additionally, RT3D demonstrated superior correlation with MRI and computed tomography compared to traditional 1D and 2D echocardiography in healthy dogs.^6^, ^25^ Therefore, RT3D served as the practical reference standard to obtain a large sample size. However, both human and several veterinary studies have found that RT3D typically underestimates volumes compared to MRI or computed tomography,^15^, ^25^, ^32^ and our RT3D results also produced the lowest recorded volumes. Furthermore, RT3D suffers from poor spatial and temporal resolution and limited imaging windows. In particular, the 4D Auto LVQ module is restricted to images acquired from the low‐frequency 4Vc‐4D transducer, which produces suboptimal resolution in smaller dogs.

Another limitation was the overrepresentation of MMVD Stage B1 dogs, as this was the most common presenting stage and the other stages, particular Stages C and D, were more easily excluded because of our medication and sedation criteria. The major criticism of 1D and 2D methods is their reliance on geometric assumptions, which changes with dilatation. Including more Stage B2, C and D dogs, such as through stratified recruitment, could have provided additional dilated hearts and influenced the results. Nevertheless, our 1D methods already had more pronounced positive proportional bias (larger biases at larger volumes) than the 2D or RT3P methods, as seen on the Bland‐Altman plots in Figures S2 and S3.

One whippet dog was excluded because of its unusually large LV volume relative to body size. Because of their distinctive body conformation and degree of athleticism, sighthound breeds normally have enlarged hearts that necessitate breed‐specific reference ranges.^34^, ^35^ Including this whippet would have introduced substantial sample variation and significantly skewed indices that assess measurement variability relative to sample variability. For example, the intraclass correlation coefficient for iESV would have increased from 0.222 to 0.658 for RT3P and from 0.198 to 0.684 for RT3D, representing a greater than 0.40 (40%) difference with the addition of just 1 dog. Consequently, the decision was made to exclude the whippet dog from all analyses, though this also limits the generalizability of the results on disproportionately large volumes relative to body size.

This study had several other limitations. First, heart rates were not standardized, which may have affected bias and agreements. Second, M‐mode measurements were timed based on the ECG rather than the position of the mitral valve, which may have negatively impacted the short‐axis 1D methods. M‐mode was chosen over B‐mode for measuring the short‐axis linear LV diameter because it is the standard method used by our clinicians. Third, the measurements varied by 3 to 5 cycles at the discretion of the operator. However, this was elected as it mimicked the authors' current clinical practice. Fourth, RT3D volume was calculated via a semi‐automated module specific to the GE system, so results may differ with other ultrasound systems and software. Fifth, the resident had different baseline experience with the 1D and 2D methods compared to the RT3P and RT3D methods, complicating conclusions about learning curves between the 2 sets. Sixth, simultaneous availability of both operators was limited within a 1‐year recruitment time frame (constrained by residency duration). A larger sample size, especially with more dilated hearts, would have provided greater statistical power and better comparisons of interoperator reproducibility. Additional operators, especially inexperienced ones for when the resident was absent, could have increased the number of operators and dogs, but this still had limited value for RT3P and RT3D as no other clinician routinely uses or was trained in these modalities. Seventh, the study involved only 2 operators from the same institution, and the resident was trained by the cardiologist for RT3P and RT3D. These factors likely artificially lowered variability. Given the lack of sedation, a limit of 2 complete echocardiograms per dog was set to minimize stress. Ideally, different operators from different institutions would be included, but this was impractical as all operators were required to obtain their own images.

In conclusion, incorporating additional dimensions appears to improve approximation of RT3D LV volume in dogs with MMVD. Although imaging view did not significantly alter bias and agreement for a given method, operators should adhere to the same imaging views. Greater skill and experience are likely needed to improve reproducibility for RT3P and RT3D among different operators.

## CONFLICT OF INTEREST DECLARATION

Authors declare no conflict of interest.

## OFF‐LABEL ANTIMICROBIAL DECLARATION

Authors declare no off‐label use of antimicrobials.

## INSTITUTIONAL ANIMAL CARE AND USE COMMITTEE (IACUC) OR OTHER APPROVAL DECLARATION

Approved by the Cornell University IACUC, protocol # 2022‐0218.

## HUMAN ETHICS APPROVAL DECLARATION

Authors declare human ethics approval was not needed for this study.

## Supporting information


**Figure S1:** Multi‐beat acquisition of RT3D images. The 3 left apical long‐axis views (4‐chamber, 2‐chamber, and 3‐chamber) are visible along the left side of the figure. Each line (both solid and dotted) in the long‐axis views represents the level from which the short‐axis views are displayed. For example, the top short‐axis views are derived from the most apical solid line, the middle short‐axis views are derived from the middle dotted line, and the bottom short‐axis views are derived from the most basilar solid line.

**Figure S2:** Bland‐Altman plots comparing all 1D, 2D, and RT3P methods with RT3D for iEDV. The line of equality (horizontal solid line), regression line of differences (alternating thick and thin dotted line), limits of agreement (horizontal thick dotted lines), and their 95% confidence intervals are displayed.

**Figure S3:** Bland‐Altman plots comparing all 1D, 2D, and RT3P methods with RT3D for iESV. The line of equality (horizontal solid line), regression line of differences (alternating thick and thin dotted line), limits of agreement (horizontal thick dotted lines), and their 95% confidence intervals are displayed.

**Table S1:** Bias and agreement of indexed LV volumes for each 1D, 2D, and RT3P echocardiographic method compared to RT3D.

**Table S2:** Intraclass correlation coefficients and reproducibility coefficients of all echocardiographic methods.

**Video S1:** Measurement of RT3D volume using the 4D Auto LVQ module. Minimal foreshortening is observed in order to highlight classic long‐axis 4‐chamber, 2‐chamber, and 3‐chamber views. The endocardial tracing is only minorly adjusted for time purposes.
