# Supplementary figures and images for: Effect of additional dimensions and views in the echocardiographic determination of 3‐dimensional left ventricular volume in myxomatous mitral valve disease in dogs
Source: J Vet Intern Med. 2025 Jan 11;39(1):e17300. doi: 10.1111/jvim.17300 (PMC11760142; doi:10.1111/jvim.17300)

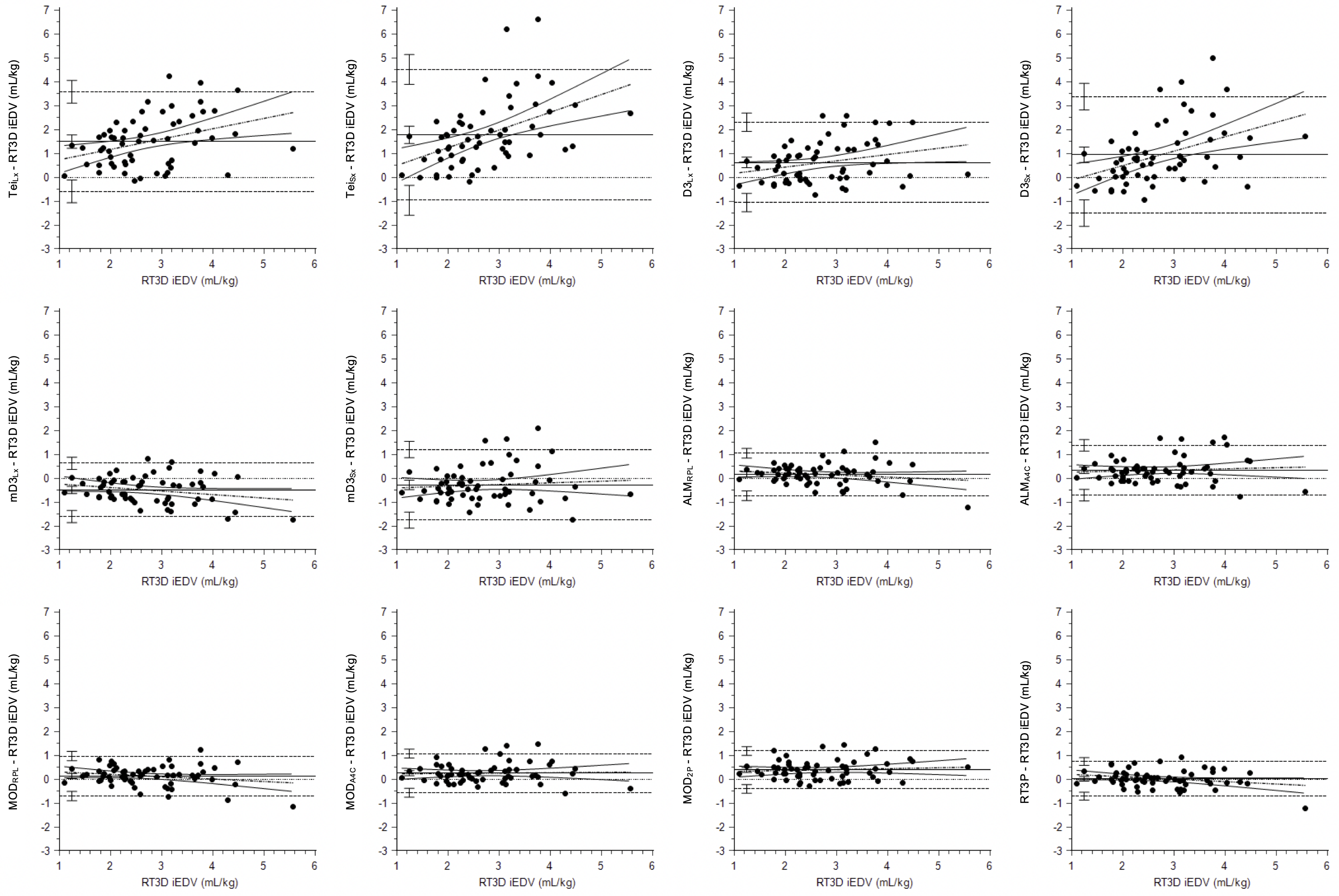

Supplement: Supplementary file 2 — Figure S2: Bland‐Altman plots comparing all 1D, 2D, and RT3P methods with RT3D for iEDV. The line of equality (horizontal solid line), regression line of differences (alternating thick and thin dotted line), limits of agreement (horizontal thick dotted lines), and their 95% confidence intervals are displayed. [file JVIM-39-e17300-s003.tiff]

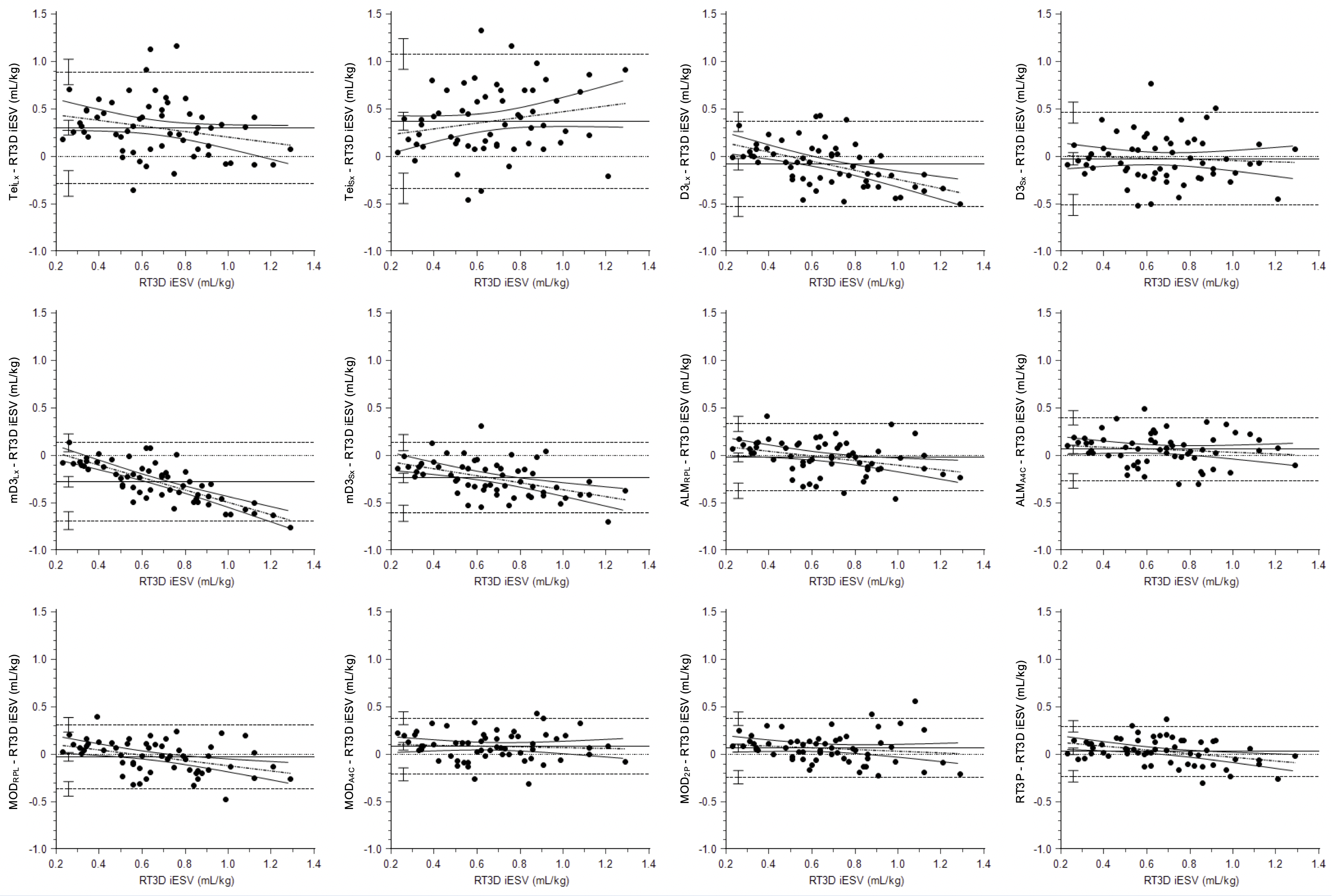

Supplement: Supplementary file 3 — Figure S3: Bland‐Altman plots comparing all 1D, 2D, and RT3P methods with RT3D for iESV. The line of equality (horizontal solid line), regression line of differences (alternating thick and thin dotted line), limits of agreement (horizontal thick dotted lines), and their 95% confidence intervals are displayed. [file JVIM-39-e17300-s005.tiff]
