# Supplementary material for: Effect of additional dimensions and views in the echocardiographic determination of 3‐dimensional left ventricular volume in myxomatous mitral valve disease in dogs
Source: J Vet Intern Med. 2025 Jan 11;39(1):e17300. doi: 10.1111/jvim.17300 (PMC11760142; doi:10.1111/jvim.17300)
Supplement: Supplementary file 4 — Table S1: Bias and agreement of indexed LV volumes for each 1D, 2D, and RT3P echocardiographic method compared to RT3D. [file JVIM-39-e17300-s006.docx]

**Supplemental Table 1 – Bias and agreement of indexed LV volumes for each 1D, 2D, and RT3P echocardiographic method compared to RT3D.**

|  |  | **iEDV** | | | | | **iESV** | | | | |
| --- | --- | --- | --- | --- | --- | --- | --- | --- | --- | --- | --- |
|  |  | **Differences** | | **Concordance Correlation** | | | **Differences** | | **Concordance Correlation** | | |
| **Method** | | **Median**  **(95% CI)** | **2.5^th^-97.5^th^ percentile** | **r_c_**  **(95% CI)** | **r_p_** | **C_b_** | **Median**  **(95% CI)** | **2.5^th^-97.5^th^ percentile** | **r_c_**  **(95% CI)** | **r_p_** | **C_b_** |
| **1D** | **Tei_Lx_** | 1.50  (1.13 – 1.73) | -0.04 – 3.99 | 0.400  (0.283 – 0.505)^a^ | 0.791 | 0.505 | 0.30  (0.22 – 0.40) | -0.18 – 1.12 | 0.328  (0.172 – 0.468)^a^ | 0.524 | 0.627 |
|  | **Tei_Sx_** | 1.60  (1.25 – 1.88) | -0.01 – 6.20 | 0.349  (0.243 – 0.447)^b^ | 0.786 | 0.445 | 0.35  (0.20 – 0.46) | -0.36 – 1.16 | 0.377  (0.247 – 0.494)^b^ | 0.679 | 0.555 |
|  | **D3_Lx_** | 0.49  (0.16 – 0.87) | -0.52 – 2.59 | 0.639  (0.512 – 0.739)^a,b,c^ | 0.810 | 0.789 | -0.06  (-0.19 – 0.01) | -0.47 – 0.42 | 0.531  (0.332 – 0.685)^c^ | 0.562 | 0.945 |
|  | **D3_Sx_** | 0.76  (0.42 – 0.94) | -0.61 – 4.02 | 0.509  (0.386 – 0.615)^d^ | 0.787 | 0.647 | -0.06  (-0.12 – 0.04) | -0.50 – 0.52 | 0.657  (0.501 – 0.772)^a,b,d^ | 0.691 | 0.952 |
|  | **mD3_Lx_** | -0.39  (-0.75 – -0.22) | -1.68 – 0.68 | 0.712  (0.581 – 0.807)^a,b,e^ | 0.810 | 0.880 | -0.24  (-0.35 – -0.19) | -0.63 – 0.08 | 0.269  (0.145 – 0.384)^d,e^ | 0.560 | 0.480 |
|  | **mD3_Sx_** | -0.44  (-0.59 – -0.19) | -1.43 – 1.65 | 0.729  (0.603 – 0.819)^a,b,f^ | 0.787 | 0.927 | -0.22  (-0.32 – -0.15) | -0.54 - 0.13 | 0.463  (0.313 – 0.591)^f^ | 0.693 | 0.669 |
| **2D** | **ALM_RPL_** | 0.22  (0.08 – 0.30) | -0.72 – 1.15 | 0.862  (0.781 – 0.915)^a,b,c,d^ | 0.878 | 0.982 | -0.01  (-0.07 – 0.07) | -0.40 – 0.34 | 0.747  (0.609 – 0.841)^a,b,e,f^ | 0.749 | 0.997 |
|  | **ALM_A4C_** | 0.30  (0.21 – 0.42) | -0.55 – 1.70 | 0.804  (0.705 – 0.872)^a,b,d^ | 0.869 | 0.925 | 0.07  (0.03 – 0.13) | -0.30 – 0.39 | 0.778  (0.660 – 0.859)^a,b,e,f^ | 0.808 | 0.964 |
|  | **MOD_RPL_** | 0.17  (0.09 – 0.26) | -0.86 – 0.85 | 0.876  (0.802 – 0.924)^a,b,c,d^ | 0.888 | 0.987 | -0.02  (-0.07 – 0.05) | -0.33 – 0.24 | 0.752  (0.617 – 0.844)^a,b,e,f^ | 0.757 | 0.994 |
|  | **MOD_A4C_** | 0.21  (0.12 – 0.32) | -0.38 – 1.40 | 0.868  (0.795 – 0.917)^a,b,c,d^ | 0.909 | 0.956 | 0.09  (0.04 – 0.13) | -0.23 – 0.37 | 0.801  (0.696 – 0.872)^a,b,c,e,f^ | 0.850 | 0.943 |
|  | **MOD_2P_** | 0.38  (0.25 – 0.49) | -0.22 – 1.38 | 0.835  (0.754 – 0.892)^a,b,c,d^ | 0.918 | 0.910 | 0.07  (0.03 – 0.11) | -0.21 – 0.41 | 0.797  (0.686 – 0.871)^a,b,c,e,f^ | 0.827 | 0.963 |
| **RT3P** | | 0.00  (-0.07 – 0.09) | -0.58 – 0.75 | 0.913  (0.858 – 0.947)^a,b,c,d,e,f^ | 0.914 | 0.999 | 0.03  (0.00 – 0.07) | -0.26 – 0.29 | 0.842  (0.749 – 0.902)^a,b,c,e,f^ | 0.851 | 0.989 |
| Abbreviations: C_b_, bias correction factor; CI, confidence intervals, r_c_, concordance correlation coefficient; r_p_, Pearson correlation coefficient  ^a^Significantly different from Tei_Lx_  ^b^Significantly different from Tei_Sx_  ^c^Significantly different from D3_Lx_  ^d^Significantly different from D3_Sx_  ^e^Significantly different from mD3_Lx_  ^f^Significantly different from mD3_Sx_ | | | | | | | | | | | |
