# Supplementary material for: Effect of additional dimensions and views in the echocardiographic determination of 3‐dimensional left ventricular volume in myxomatous mitral valve disease in dogs
Source: J Vet Intern Med. 2025 Jan 11;39(1):e17300. doi: 10.1111/jvim.17300 (PMC11760142; doi:10.1111/jvim.17300)
Supplement: Supplementary file 5 — Table S2: Intraclass correlation coefficients and reproducibility coefficients of all echocardiographic methods. [file JVIM-39-e17300-s004.docx]

**Supplemental Table 2 – Intraclass correlation coefficients and reproducibility coefficients of all echocardiographic methods.**

|  |  | **iEDV** | | **iESV** | |
| --- | --- | --- | --- | --- | --- |
| **Method** | | **ICC**  **(95% CI)** | **RC (mL/kg)**  **(95% CI)** | **ICC**  **(95% CI)** | **RC (mL/kg)**  **(95% CI)** |
| **1D** | **Tei_Lx_** | 0.933  (0.862 – 0.969) | 1.27  (1.01 – 1.71) | 0.699  (0.452 – 0.848) | 0.67  (0.53 – 0.90)^a^ |
|  | **Tei_Sx_** | 0.904  (0.778 – 0.957) | 1.79  (1.42 – 2.42)^b^ | 0.838  (0.679 – 0.922) | 0.58  (0.46 – 0.79)^b^ |
|  | **D3_Lx_** | 0.911  (0.818 – 0.958) | 1.26  (1.00 – 1.70) | 0.671  (0.409 – 0.832) | 0.47  (0.37 – 0.63)^c^ |
|  | **D3_Sx_** | 0.883  (0.744 – 0.946) | 1.78  (1.41 – 2.40)^d^ | 0.826  (0.657 – 0.915)^d^ | 0.42  (0.33 – 0.56)^d^ |
|  | **mD3_Lx_** | 0.911  (0.818 – 0.958) | 0.84  (0.67 – 1.14)^b,d,e^ | 0.669  (0.406 – 0.831) | 0.32  (0.25 – 0.43)^a,b,e^ |
|  | **mD3_Sx_** | 0.883  (0.743 – 0.946) | 1.19  (0.94 – 1.61) | 0.827  (0.660 – 0.916)^f^ | 0.28  (0.22 – 0.37)^a,b,f^ |
| **2D** | **ALM_RPL_** | 0.803  (0.559 – 0.911) | 1.38  (1.10 – 1.87)^g^ | 0.461  (-0.025 – 0.746) | 0.92  (0.72 – 1.23)^c,d,e,f^ |
|  | **ALM_A4C_** | 0.821  (0.622 – 0.916) | 1.46  (1.15 – 1.98)^e,h^ | 0.319  (-0.102 – 0.651)^d,f^ | 1.09  (0.86 – 1.47)^b,c,d,e,f,h^ |
|  | **MOD_RPL_** | 0.894  (0.480 – 0.965) | 0.97  (0.77 – 1.31)^b,d^ | 0.615  (-0.032 – 0.856) | 0.61  (0.49 – 0.83)^e,f,h,i^ |
|  | **MOD_A4C_** | 0.925  (0.838 – 0.966) | 0.82  (0.65 – 1.11)^b,d,h,j^ | 0.431  (-0.058 – 0.731) | 0.82  (0.65 – 1.10)^c,d,e,f^ |
|  | **MOD_2P_** | 0.942  (0.872 – 0.973) | 0.78  (0.62 – 1.06)^b,d,g,h,k^ | 0.430  (0.011 – 0.707) | 0.83  (0.66 – 1.12)^c,d,e,f^ |
| **RT3P** | | 0.860  (0.545 – 0.946) | 1.08  (0.86 – 1.47) | 0.222  (-0.099 – 0.536)^d,f^ | 1.29  (1.02 – 1.74)^a,b,c,d,e,f,i^ |
| **RT3D** | | 0.771  (0.113 – 0.924) | 1.54  (1.22 – 2.08)^e,h,j,k^ | 0.198  (-0.100 – 0.510)^d,f^ | 1.31  (1.04 – 1.77)^a,b,c,d,e,f,i^ |
| Abbreviations: CI, confidence intervals; ICC, intraclass correlation coefficient; RC, reproducibility coefficient  ^a^Significantly different from Tei_Lx_  ^b^Significantly different from Tei_Sx_  ^c^Significantly different from D3_Lx_  ^d^Significantly different from D3_Sx_  ^e^Significantly different from mD3_Lx_  ^f^Significantly different from mD3_Sx_  ^g^Significantly different from ALM_RPL_  ^h^Significantly different from ALM_A4C_  ^i^Significantly different from MOD_RPL_  ^j^Significantly different from MOD_A4C_  ^k^Significantly different from MOD_2P_ | | | | | |
